# ADHD Reporting in Developmental Age: The Role of the Informants

**DOI:** 10.3390/children12070914

**Published:** 2025-07-11

**Authors:** Dario Esposito, Federica Gigliotti, Beatrice Colotti, Carlo Di Brina, Francesco Pisani, Maria Romani

**Affiliations:** Department of Human Neuroscience, Sapienza University of Rome, 00185 Roma, Italy; federica.gig@uniroma1.it (F.G.); carlo.dibrina@uniroma1.it (C.D.B.); francesco.pisani@uniroma1.it (F.P.); maria.romani@uniroma1.it (M.R.)

**Keywords:** ADHD, informants, school reporting, developmental age

## Abstract

**Background:** Attention-Deficit/Hyperactivity Disorder (ADHD) is a complex neurodevelopmental condition typically requiring information from multiple informants for accurate diagnosis. However, the consistency and diagnostic value of reports from teachers, parents, primary care providers (PCPs), and other professionals remain debated. This study aimed to examine the role and diagnostic accuracy of different informants in the referral and diagnostic process for ADHD in children aged 3–11. **Methods**: This retrospective study analyzed data from 120 children referred for suspected ADHD. Initial reports were obtained from teachers, parents, PCPs, and other professionals, and final diagnoses were determined through comprehensive neuropsychiatric evaluations. Diagnostic concordance and informant-specific contributions were assessed. **Results**: Of the 120 children, 64 (53.3%) received an ADHD diagnosis. Teachers were the most frequent informants, followed by parents, with fewer referrals from PCPs and other professionals. No significant differences in diagnostic accuracy were found among informants, aligning with previous studies suggesting that no single informant is superior in identifying ADHD. Notably, over 93% of referred children were diagnosed with a neuropsychiatric disorder, though not necessarily ADHD. **Conclusions**: The findings underscore the importance of combining reports from parents and teachers to capture symptom expression across different environments, which is essential for accurate ADHD diagnosis. Enhanced training for informants and a multidisciplinary approach is recommended to improve diagnostic accuracy and support early identification and intervention efforts. These results support nuanced evaluation strategies that account for informant variability and help mitigate potential misinterpretations of ADHD symptoms.

## 1. Introduction

Attention-Deficit/Hyperactivity Disorder (ADHD) is a persistent and pervasive neurodevelopmental disorder. It is characterized by levels of hyperactivity, impulsivity, and/or inattention that are inappropriate for an individual’s developmental stage. These symptoms can lead to impairments in daily functioning, particularly in areas such as academic performance, health, and social interactions. ADHD is typically diagnosed in childhood, with reported prevalence rates varying widely from 1.5% to 19.9%, though the average is approximately 5% [1,2].

The diagnosis of ADHD is complex, requiring information from multiple sources and clinical observation. Specifically, the presence of symptoms must be confirmed in at least two different settings. Parents and teachers are the most common informants, as they often have a deeper understanding of a child’s daily functioning. However, pediatricians, general practitioners, and other professionals are also frequently involved in the referral process [1].

While the involvement of multiple informants is necessary for a thorough assessment, it can also result in discrepancies in the evaluation of a child’s behavior. A frequently debated question in the literature is whether some informants are more reliable than others in the referral process for ADHD [3,4].

Discrepancies between informants are common in the assessment of child and adolescent psychopathology. Theoretical models such as the multi-informant model [5,6] suggest that differences in symptom reporting are not merely due to measurement error, but may reflect meaningful contextual variability—that is, variation in behavior across home, school, or clinical environments—as well as source-specific biases linked to the informant’s perspective, expectations, or relationship with the child [7,8]. For instance, teachers may be more sensitive to attentional difficulties in structured settings, while parents might be better at detecting emotional or internalizing symptoms. These differences underscore the importance of integrating multiple perspectives during clinical evaluation, especially in conditions like ADHD, where symptom expression is highly context-dependent. Despite widespread recommendations for a multi-informant approach, few studies have assessed the comparative clinical validity of different informant types within real-world referral settings [5,6,7]. To our knowledge, this is the first study to compare, in a real clinical setting, the referrals for inattention and/or hyperactivity/impulsivity made by various informants with the final diagnoses established after a comprehensive diagnostic evaluation. This approach aims to highlight differences in assessments among various informants, as well as to identify the strengths and weaknesses of each informant type.

## 2. Materials and Methods

This study employed a retrospective, non-interventional design, analyzing data from patients aged three to eleven years who were referred to our child neuropsychiatry secondary care center in Central Italy. We included all children who were present for their first clinical examination at our center between January 2021 and June 2022. Following the initial visit, only those children who were registered with a primary complaint of symptoms related to hyperactivity/impulsivity and/or inattention were included for further analysis. For these patients, data were then collected regarding the individual or entity that initiated the referral for evaluation, whether it was an independent decision made by the parents or a referral suggested by another person familiar with the child (e.g., a teacher, pediatrician, or other professional). Patients seeking a second opinion, as well as those with pre-existing diagnoses of intellectual disability, autism spectrum disorder, or other complex neurodevelopmental disabilities, were excluded from the study.

Our analysis focused on several key aspects: the identification of the informants who initiated the clinical evaluation (e.g., teachers, pediatricians, or parents); the reasons for referral (i.e., hyperactivity/impulsivity, inattention, or a combination of these symptoms); and a comparison of the initial reports with the final diagnoses obtained after a standard comprehensive diagnostic evaluation.

All children underwent a comprehensive neuropsychiatric assessment conducted by a multidisciplinary team including child neuropsychiatrists and psychologists. The final diagnosis was based on DSM-5 criteria. Standardized instruments were employed, including parent- and teacher-rated ADHD symptom questionnaires, checklists for internalizing and externalizing symptoms, cognitive evaluations, and assessments of executive functioning, language development, academic skills, and graphomotor abilities.

Continuous variables are presented using the mean ± standard deviation (SD) and range, while categorical variables are presented as counts and percentages. For group comparisons, we used an unpaired Welch’s *t*-test for continuous variables, selected due to the unequal variances among comparison groups. For categorical variables, either the chi-square test or Fisher’s exact test (when expected frequencies were less than 5) was used. Variables with low expected counts were dichotomized into 2 × 2 contingency tables to appropriately apply the Fisher’s exact test (e.g., smaller informant groups were combined with larger related categories to ensure statistical validity). A *p*-value of less than 0.05 was considered statistically significant for all comparisons. Descriptive and inferential analyses were conducted using IBM SPSS Statistics software (v. 30.0.0)

## 3. Results

Out of 1140 children between three and eleven years of age referred to our outpatient center for the first time between 2021 and 2022, 120 children (10.52%) met our inclusion criteria (mean age 6.83, SD: 1.82 years; 73.3% males). Table 1 summarizes the main demographic characteristics of our sample.

The children were reported by various sources: 65 by teachers (54.17%), 30 by parents (25%), 10 by both teachers and parents (8.3%), 9 by doctors (7.5%) and 6 by other professionals (5%). In Table 2 more details are reported.

The diagnostic outcomes of the patients were categorized into four groups: 14 patients with ADHD only, 50 with ADHD plus at least one comorbidity, 48 with alternative diagnoses, and 8 without any clinically relevant pathology.

Overall, 64 of the 120 referred children (53.3%) were ultimately diagnosed with ADHD. This corresponds to 5.6% of all patients seen at the outpatient clinic during the study period (*n* = 1140). Among the 64 children confirmed with ADHD, 16 were female (which is 50% of the 32 referred girls) and 48 were male (54% of the 88 referred boys). Thus, about half of the referred children of each gender received an ADHD diagnosis. An odds ratio (OR) analysis showed that the odds of a referred boy being diagnosed with ADHD were only slightly higher than that of a referred girl (OR ≈ 1.2, 95% confidence interval [0.54–2.70]), and this difference was not statistically significant (χ^2^(1) = 0.04, *p* = 0.85). In other words, the diagnostic yield was comparable for boys and girls, despite the higher number of boys referred (male-to-female ratio ≈ 2.75:1, *p* < 0.001 by binomial test).

Within the ADHD-positive group (*n* = 64), 14 children (22%) had an exclusive ADHD diagnosis (no comorbid conditions), whereas 50 children (78%) had ADHD with comorbidities. On average, children with ADHD had 1.6 comorbid diagnoses (SD = 0.6, median = 2). The mean age at ADHD diagnosis was 6.9 years. Because the age distribution approximated normal in both the “ADHD-only” and “ADHD plus” groups (Shapiro–Wilk *p* > 0.10), we used an independent samples *t*-test to compare their ages. This revealed no significant age difference between children with pure ADHD and those with ADHD plus comorbidities, with a negligible effect size (Cohen’s d 0.1). Thus, the age at diagnosis was similar regardless of the presence of comorbid conditions.

We explored whether the likelihood of an ADHD diagnosis varied by the child’s age at referral. We grouped the referred children into three age ranges (preschool: 3–5 years; early school age: 6–7 years; and later childhood: 8–11 years). There was no statistically significant difference in ADHD diagnostic rates across these age groups (χ^2^(2) = 5.23, *p* = 0.07), although there was a trend suggesting a higher yield in the early school-age group.

Most of the girls reported with a hyperactive or combined presentation were diagnosed with ADHD (respectively, 74 and 72%), while most of those reported with inattentive symptoms had other comorbidities (29% with ADHD). In boys, the results were more homogenous, with about 50% of those reported with inattentive or hyperactive symptoms being diagnosed with ADHD, and 59% of those reported with combined symptoms receiving a diagnosis.

ADHD comorbidities were further divided into two groups: emotional–behavioral disorders (22 children, with a 3:1 ratio between internalizing [e.g., depression] and externalizing disorders [e.g., oppositional defiant disorder]) and other neurodevelopmental disorders (42 children, including specific learning disorder [SLD]—which was the most frequent—low intellectual functioning, and autism spectrum disorders). Fifteen children had co-occurring conditions and belonged in both groups.

Probands without ADHD can be further classified into two subsets: 48 had other diagnoses (with a mean number of disorders of 1.7; SD 0.94), and in 8 there was no clinically relevant condition (see Table 2). Among these children, 38 were diagnosed with emotional–behavioral disorders (with a ratio of approximately 2:1 between internalizing and externalizing disorders), while 27 had other neurodevelopmental disorders (with SLD being the most frequent in this group as well); 17 children had both diagnoses.

There were no statistically significant differences in ADHD diagnoses across age groups, although a trend was observed (*p* = 0.07), with most children with confirmed ADHD being referred in the age range between 6 and 7 years.

There was a significantly higher number of referrals for males compared to females (*p* < 0.01); however, no significant differences were found in the proportions of ADHD diagnoses between males and females (*p* = 0.85). Notably, symptom presentation was associated with the likelihood of an ADHD diagnosis (*p* < 0.01 for group differences). Children referred for combined-type symptoms had the highest confirmation rate of ADHD (62.5% were diagnosed), followed by those with predominantly hyperactive–impulsive symptoms (55.6% diagnosed), while those with predominantly inattentive symptoms had the lowest ADHD confirmation rate (44.4% diagnosed). The overall association between reported symptom type and diagnostic outcome corresponded to a small-to-moderate effect size (Cramér’s V ≈ 0.17; equivalent to Cohen’s *h* ≈ 0.36 for the proportion difference between the highest and lowest groups), indicating that the differences, although statistically significant, were modest in magnitude. A post hoc power analysis for this comparison yielded an approximate power of 52%, suggesting a moderate ability to detect these differences given our sample size.

In our sample, the most frequently involved informants were teachers (57.7% of referrals) (*p* < 0.01), followed by parents (30.8%), while only a small percentage of children were referred by other professionals: primary care practitioners (PCPs), speech therapists, and psychologists. Table 2 provides detailed information about informants. It should be considered that 10 children were reported jointly by both parents and teachers.

In our sample, about half of patients reported for suspected ADHD by teachers (56%) or parents (52.5%) received a confirmed diagnosis of ADHD after a complete diagnostic evaluation. There was no correlation between the type of signaler and the likelihood of a correct ADHD diagnosis (*p* = 0.62). In particular, a chi-square test comparing diagnostic outcomes across the referral groups yielded χ^2^(4) = 3.27, *p* = 0.514. The corresponding effect size was small (Cramér’s V = 0.165), with an estimated statistical power of 26%, indicating a limited ability to detect subtle differences across informants.

## 4. Discussion

This study explored the relationship between reports of ADHD symptoms by schools, families, and other professionals, and the final diagnoses of ADHD.

The initial sample of children suspected of inattention/hyperactivity presented with epidemiological characteristics that are consistent with the previous literature. A large majority of the children reported were indeed male (73.3%) [9,10]; most of the girls in our sample were reported for inattention, which is in line with the finding that girls are less likely to be reported for overt symptoms (hyperactivity/impulsivity) [11]. Moreover, most of the reported children were between 6 and 7 years old, probably because this is the age at which Italian children usually enter primary school, and, therefore, ADHD symptoms may become incompatible with the tasks required in a school context. In older age groups, the number of reports decreases (47% of reports at 6–7 years vs. 34% in the older age group), which is also probably associated with the gradual reduction in the more overt symptoms [12]. In accordance with this, inattention was a slightly more common reason for reporting, especially in the female group (53% of reports) and in the older age groups (49% of reports).

Children were primarily referred by teachers, with a considerably smaller proportion referred by parents (approximately 58% versus 31%). Only 8.3% of cases involved joint reporting by both parents and teachers, highlighting a low level of collaboration and shared concern between these two groups.

Teachers are widely regarded as a crucial source of information in assessing ADHD symptoms in children [13]. According to the literature, approximately 85% of clinicians rely on teacher-administered questionnaires to evaluate ADHD symptoms within the school context [13]. Several authors emphasize the value of teachers’ evaluations, noting that their experience with age-appropriate behavior often aligns more closely with a clinician’s perspective [7,13].

Parental reporting is equally important, as it captures the child’s behavior in everyday life and provides a detailed account of their developmental history. However, parental reporting is not without limitations and biases, including a tendency to underestimate ADHD symptoms, particularly in preschool-aged children and girls [14].

A small minority of referrals in our study came from primary care providers (PCPs), such as pediatricians (6.9%), and other professionals, including psychologists, developmental therapists, and speech therapists (4.6%). PCPs play a pivotal role in ensuring appropriate referrals, acting as an essential clinical “filter” between the child, other informants, and specialized psychiatric services.

Nevertheless, PCPs face several challenges in the referral process, often stemming from a lack of specific knowledge about ADHD, misjudgments or biases regarding the disorder, and limited resources to support patients effectively [15]. International studies indicate that the percentage of PCPs who have received formal education on ADHD during their undergraduate and postgraduate training ranges from only 1% to 28% of physicians [15]. This underscores the importance of targeted educational programs, which are proven to be effective in increasing awareness and fostering a greater confidence in diagnosing and managing ADHD [16].

Another significant obstacle reported by PCPs is the scarcity of resources needed for ADHD assessments, particularly in time-constrained settings. The accurate evaluation of this multifaceted condition requires a multidisciplinary, thorough, and time-intensive diagnostic approach [16].

Among the 1140 children referred to our center, 5.6% were ultimately diagnosed with ADHD. Although influenced by referral bias, this rate is higher than the reported national average for Italy (1.1–3.1%) [17], yet consistent with the global prevalence estimates of approximately 5% [1].

Following diagnostic evaluations, the male predominance observed in initial referrals was confirmed, with boys comprising 75% of the ADHD cases. Children in the youngest age group (3–5 years) were more frequently referred by teachers (*p* < 0.01). Additionally, ADHD diagnoses accompanied by comorbidities showed a strong association with neurodevelopmental disorders, primarily learning disabilities. This finding aligns with the existing literature, which identifies ADHD as the most common comorbidity in patients with specific learning disorders (SLD); both conditions involve deficits in executive functioning [18].

Children who were “erroneously” referred for ADHD were most commonly diagnosed with emotional–behavioral disorders, with no significant gender differences. Only 30% of these children received a diagnosis of a neurodevelopmental condition, such as a specific learning disorder (SLD), without co-occurring emotional–behavioral disorders. While conditions like oppositional defiant disorder (ODD) and other externalizing disorders frequently co-occur with ADHD, they can also mimic hyperactive behaviors [19]. Additionally, anxiety and depressive symptoms in childhood can resemble both inattentive and hyperactive traits associated with ADHD [20,21].

The ratio of confirmed ADHD diagnoses to initial suspicions might suggest that parents and teachers have statistically similar “reporting abilities.” Chi-square analysis revealed no significant differences in diagnostic confirmation rates among cases referred by teachers, parents, or other professionals. This aligns with the existing literature suggesting that no single group of informants is definitively superior in accurately identifying children with suspected ADHD [6,21]. Research indicates that both parents and teachers exhibit a moderate to good diagnostic accuracy for ADHD, with no significant differences in their predictive ability for a gold-standard ADHD diagnosis [22,23]. However, it should be noted that the small observed effect size and limited statistical power imply that these conclusions must be interpreted with caution. The current sample may not have been large enough to detect subtle but clinically meaningful differences between informant groups. Therefore, further research with larger and more statistically powered samples will be necessary to draw more definitive conclusions about the relative value of each informant type in the diagnostic process.

No single group of informants in our sample proved to be entirely reliable, and inconsistencies were observed across all sources. Despite teachers generating a greater number of referrals, the actual ADHD diagnosis rate remained around 50%, even when stratified by gender or age. This finding challenges the notion that specific reporters are markedly more accurate and is consistent with the recent literature on this topic [24]. However, earlier studies suggest that teacher ratings may surpass parent ratings in terms of sensitivity, specificity, and overall classification accuracy [6]. This could be attributed to the teachers’ greater experience in distinguishing age-appropriate behaviors within a school context.

Importantly, due to the retrospective nature of the sample, socio-economic status, ethnicity, and parental education level were not consistently recorded and could not be included in the analysis. We acknowledge this as a significant limitation, as such variables may influence both the behavior of the informants and the clinical presentation of the child. Moreover, the retrospective design inherently introduces a potential referral bias, as the sample was composed exclusively of children who had already been referred for concerns related to attention, behavior, or development. This may limit the representativeness of the findings compared to community-based or screening populations. Additionally, the study was conducted in a single Italian tertiary center, which may affect the generalizability of results to other geographic, cultural, or clinical settings. Future research should aim to replicate these findings in multi-center and prospective samples, ideally incorporating systematic socio-demographic data collection.

Discrepancies between informants are often shaped by two interacting mechanisms: source bias—that is, systematic distortions in the informants’ reports based on their expectations, roles, or relationships with the child—and contextual variability, referring to genuine differences in behavior across settings such as the home and school [4,5,8]. These concepts are well established in the multi-informant literature and suggest that the disagreement between sources does not necessarily imply error or noise but may convey clinically meaningful information.

Recent models [8] propose subtyping ADHD presentations based on the context in which symptoms manifest—such as school-only, home-only, or pervasive—arguing that context-specific patterns may have different prognostic and functional implications. Similarly, the “satellite model” described by De Los Reyes et al. [25] emphasizes the value of integrating information from multiple informants not by averaging responses but by recognizing the unique perspective each source provides within its environment. This framework challenges the notion of a single “true” report and instead supports an ecologically grounded interpretation of symptom expression.

In this light, discrepancies between parents and teachers—as observed in our study—may not undermine diagnostic validity but reflect the situational nature of ADHD symptomatology. Clinical practice should therefore move beyond a simplistic reconciliation of differences and toward an informed integration of diverse, context-sensitive data sources.

This dual-informant (parent–teacher) approach enhances the diagnostic process by capturing symptoms across different environments, a critical factor in accurately diagnosing ADHD [23].

However, it should be noted that more than 93% of patients referred for suspected ADHD were ultimately diagnosed with a neuropsychiatric disorder, even if it was not ADHD. This suggests that informants may not necessarily be misreporting in an absolute sense but may instead misinterpret symptoms and categorize them incorrectly. This underscores the value of the reporters’ perspectives and emphasizes the need for a more nuanced approach to diagnosis.

These findings highlight relevant practical and policy implications. Firstly, the comparable diagnostic accuracy observed across informants reinforces the importance of systematically integrating both teacher and parent reports during the assessment process. Clinical procedures should include structured multi-informant screening tools to capture symptom consistency across different contexts. Secondly, given the teachers’ prominent role in referrals, targeted training programs to enhance teachers’ ability to identify ADHD symptoms, especially in under-reported groups such as girls with inattentive presentations, are recommended. Lastly, strengthening collaboration between schools, primary care providers, and specialized services can help streamline referrals, reduce diagnostic delays, and promote timely interventions through clear, multidisciplinary referral pathways.

## Figures and Tables

**Table 1:** Demographic data of reported children by age group, gender, and ADHD symptom presentation. ^1^: percentage within the respective age group or gender; ^2^: percentage of the total sample.

Reported Children	Predominant Inattentive Symptoms *n* (%)	Predominant Hyperactive/Impulsive Symptoms *n* (%)	Combined Symptoms *n* (%)	Total *n* (%)
**Age Groups**				
3–5 years old	8 (36.4) ^1^	4 (18.2) ^1^	10 (45.4) ^1^	22 (18.3) ^2^
6–7 years old	26 (45.6) ^1^	11 (19.3) ^1^	20 (35.1) ^1^	57 (47.5) ^2^
8–11 years old	20 (48.8) ^1^	3 (7.3) ^1^	18 (43.9) ^1^	41 (34.2) ^2^
**Gender**				
Male	37 (42.0) ^1^	14 (16.0) ^1^	37 (42.0) ^1^	88 (73.3) ^2^
Female	17 (53.1) ^1^	4 (12.5) ^1^	11 (34.4) ^1^	32 (26.7) ^2^
Total	54 (45.0) ^2^	18 (15.0) ^2^	48 (40.0) ^2^	120 (100) ^2^
	**3–5 years old ** ***n *** **(%)**	**6–7 years old ** ***n *** **(%)**	**8–11 years old ** ***n *** **(%)**	**Total ** ***n *** **(%)**
Male	18 (81.8) ^1^	42 (73.7) ^1^	28 (68.3) ^1^	88 (73.3) ^2^
Female	4 (18.2) ^1^	15 (26.3) ^1^	13 (31.7) ^1^	32 (26.7) ^2^
Total	22 (18.3) ^2^	57 (47.5) ^2^	41 (34.2) ^2^	120 (100) ^2^

**Table 2:** Epidemiological and clinical data of subjects categorized by informants. Percentages refer to the respective age group, gender, reason for referral, or diagnosis. PCPs: Primary Care Practitioners. Note: The total number of children in these categories may exceed 100% (*n = *120) because 8.3% of the children (*n = *10) were referred by both teachers and parents and are therefore included in multiple categories.

Informants	Teachers *n* (%)	Parents *n* (%)	PCPs *n* (%)	Other Professionals *n* (%)
**Age Groups**				
3–5 years old (*n = *22)	11 (50)	7 (31.8)	3 (13.6)	2 (9.1)
6–7 years old (*n =* 57)	40 (70.2)	16 (28.1)	4 (7.0)	4 (7.0)
8–11 years old (*n* = 41)	24 (58.5)	17 (41.5)	2 (4.9)	0
**Gender**				
Male (*n* = 88)	57 (64.8)	31 (35.2)	4 (4.5)	4 (4.5)
Female (*n* = 32)	18 (56.2)	9 (28.1)	5 (15.6)	2 (6.2)
**Reasons for Reporting**				
Inattentive Symptoms (*n* = 54)	33 (61.1)	15 (27.8)	6 (11.1)	3 (5.5)
Hyperactive–Impulsive (*n =* 18)	9 (50.0)	7 (38.9)	3 (16.7)	1 (5.5)
Combined (*n* = 48)	33 (68.8)	18 (37.5)	0	2 (4.2)
**Diagnosis**				
ADHD (*n* = 64)	42 (65.6)	21 (32.8)	6 (9.4)	2 (3.1)
Other Diagnosis (*n* 48)	28 (58.3)	16 (33.4)	2 (41.7)	4 (8.4)
No Diagnosis (*n* 8)	5 (6.2)	3 (3.7)	1 (1.2)	0
**Total sample**	75 (57.7)	40 (30.8)	9 (6.9)	6 (4.6)
**ADHD Diagnosis/Reported**	42/75 (56.0)	21/40 (52.5)	6/9 (66.0)	2/6 (33.3)

## Data Availability

The data presented in this study are available on request from the corresponding author. The data are not publicly available due to privacy restrictions.
